# [Retracted] Rosuvastatin suppresses platelet‑derived growth factor‑BB‑induced vascular smooth muscle cell proliferation and migration via the MAPK signaling pathway

**DOI:** 10.3892/etm.2023.12299

**Published:** 2023-11-13

**Authors:** Jianting Gan, Ping Li, Zhengdong Wang, Jian Chen, Xiangwen Liang, Ming Liu, Wenchao Xie, Ruixing Yin, Feng Huang

Exp Ther Med 6:899–903, 2013; 10.3892/etm.2013.1265

Following the publication of this paper, it was drawn to the Editors’ attention by a concerned reader that the western blotting data shown in Figs. 2 and 4, and the Transwell migration assay data in Fig. 3B, were strikingly similar to data appearing in different form in other articles written by different authors at different research institutes that had either already been published elsewhere prior to the submission of this paper to *Experimental and Therapeutic Medicine*, or were under consideration for publication at around the same time. In view of the fact that these data had already apparently been published previously, the Editor of *Experimental and Therapeutic Medicine* has decided that this paper should be retracted from the Journal. The authors were asked for an explanation to account for these concerns, but the Editorial Office did not receive a reply. The Editor apologizes to the readership for any inconvenience caused.

